# Obesity and COVID-19 in-hospital fatality in southern Brazil: impact by age and skin color

**DOI:** 10.11606/s1518-8787.2022056004329

**Published:** 2022-02-09

**Authors:** Gbènankpon Mathias Houvèssou, Daniel G P Leventhal, Eduardo Viegas da Silva

**Affiliations:** I Universidade Federal de Pelotas Faculdade de Medicina Programa de Pós-Graduação em Epidemiologia Pelotas RS Brasil Universidade Federal de Pelotas. Faculdade de Medicina. Programa de Pós-Graduação em Epidemiologia. Pelotas, RS, Brasil; II Centro de Vigilância em Saúde do Estado do Rio Grande do Sul Porto Alegre RS Brasil Centro de Vigilância em Saúde do Estado do Rio Grande do Sul. Porto Alegre, RS, Brasil

**Keywords:** COVID-19, mortality, Obesity, Race Factors, Hospitalization, Retrospective Studies

## Abstract

**OBJECTIVES:**

To estimate the relative risk (RR) of death associated with obesity, the attributable fraction in the exposed/with obesity (AF_o_), and the hospitalized population attributable risk (hospitalized PAR) associated with obesity of death among all adults and among Black and non-Black adults hospitalized for severe COVID-19 in the state of Rio Grande do Sul, Brazil.

**METHODS:**

This retrospective cohort study of prognostic factors analyzed all cases of adults hospitalized for severe COVID-19 in the state of Rio Grande do Sul, Brazil. The occurrence of obesity was measured using secondary data from hospital teams’ surveillance records. The outcome assessed was hospital deaths caused by severe COVID-19. Poisson regression was used to estimate RRs and 95% confidence intervals (95%CI).

**RESULTS:**

The study sample consisted of 100,099 patients hospitalized for severe COVID-19, most of whom were White (84.7%) and male (54.7%). The effect of obesity was strongly modified by age, being higher in younger age groups. For the 18–39-year-old age group, RR = 2.54 (95%CI: 2.33–2.77), and in individuals 70 years and above, RR = 1.09 (95%CI: 1.05–1.13). For the 18–39-year-old age range, AF_o_ = 60.6% and AF_o_ = 42.5% in individuals 40–59 years old. For all hospitalizations, Hospitalized PAR measuring obesity for individuals 18–39 years old was 25.3%, while in the 40–59-year-old range, the hospitalized PAR = 11.2%. The hospitalized PAR was 31.7% in the Black population aged 18–39 years and 24.8% in non-Blacks. The hospitalized PAR was also larger in Blacks aged 40-59 years.

**CONCLUSIONS:**

Obesity largely impacted in-hospital case-fatality rates among young adults and Black people contaminated by COVID-19. These data highlight the extent of the risk concerning obesity, a highly prevalent chronic condition.

## INTRODUCTION

Since its original recognition in the city of Wuhan, Hubei province, China, in December, 2019^1^, the new coronavirus (SARS-CoV-2) has caused an elevated incidence of COVID-19 globally with catastrophic consequences. Among countries most affected, Brazil displayed the greatest mortality rate per 100,000 worldwide, in September, 2021^2^, with 580,413 deaths as of September 1, 2021^3^.

Chronic conditions, including diabetes mellitus, cardiovascular diseases, chronic pulmonary diseases and others^4^, are prognostic factors associated with the aggravation of cases and with an outcome of death by COVID-19^5^. Obesity commonly co-occurs with such morbidities, but it also makes independent contributions in causal mechanisms of the aggravation of infectious cases and of resultant death^6^. This risk factor has become a greater concern as obesity prevalence is increasing in almost all countries, reaching a global rate of 13.2% among adults in 2019^7^ and affecting more than 650 million adults and 124 million children worldwide^8^. In Brazil, estimated prevalence in adults was 26.8% in 2019^9^.

Obesity is also increasingly being recognized as a prognostic factor for severe cases of COVID-19^10^. Studies reporting its relation with the case fatality rate from the disease reveal that obese people have a 3.04^11^, 3.78^12^, and 1.45^13^ times greater risk of death compared to non-obese people. However, some discrepancies are observed. A prospective cohort study with 1,150 adults hospitalized with COVID-19 in New York did not find evidence of obesity being an independent risk factor for hospital death^14^, and a systematic review found an association between obesity and severe cases of the disease, but not with a greater mortality^15^.

Such studies did not estimate effect modification by obesity on the hospital case-fatality rate by COVID-19 according to age^11^, and only two studies^16,17^ in the literature evaluated age as an effect modifier of obesity. Moreover, we found no studies estimating the risk of death attributable to obesity in the adult population hospitalized for COVID-19 nor in the Black and non-Black adult demographic subgroups hospitalized for COVID-19. This knowledge is important for identifying those groups for which obesity displays a greater attributable risk and for estimating its impact. Such information would improve health promotion activities aimed at vulnerable groups, especially since the prevalence of this risk factor is high and its distribution in middle-income countries^18^ is unequal.

This study aimed to estimate the relative risk associated with obesity for death among adults hospitalized for COVID-19 in the state of Rio Grande do Sul (RS), Brazil. The study also aimed to test the hypothesis of modification of the association according to age, to estimate the hospitalized population attributable risk (hospitalized PAR) with obesity, as well as that among Black and non-Black people hospitalized for COVID-19 in RS.

## METHODS

### Participants and Data Sources

This retrospective cohort study of prognostic factors analyzed cases hospitalized for severe COVID-19 of residents in the state of Rio Grande do Sul, which is located in the South of Brazil and has an estimated population of 11,466,630 inhabitants for the year 2021^19^. Secondary data of hospitalizations notified in the *Sistema de Vigilância Epidemiológica da Gripe* (SIVEP Gripe – Influenza Epidemiological Surveillance Information System) of the Ministry of Health were used. The analytical sample included confirmed hospitalizations for severe COVID-19 occurring since the beginning of the pandemic, for individuals 18 years or older, who had a recorded outcome of hospitalization (discharge or death) by 9/20/2021. Cases of severe COVID-19 were notified from public and private health facilities. Data on the origin of case notifications were obtained from the *Cadastro Nacional de Estabelecimentos de Saúde* (CNES – National Health Facility Registry) website^20^.

The primary instrument of data collection by health facility teams was the compulsory notification sheet of cases of hospitalized severe COVID-19 for the SIVEP Gripe. Cases of severe COVID-19 are defined as individuals with flu-like illness (acute respiratory illness, characterized by at least two of the following signs and symptoms: fever, chills, sore throat, headache, cough, runny nose, loss of smell or taste) who present with dyspnea/respiratory discomfort, persistent pressure in the thorax, O_2_ saturation less than 95% in ambient air, or blueness in the lips or face^21^.

### Exposure Assessed

The occurrence of obesity was primarily measured by health facility teams observing individuals’ medical records. SIVEP Gripe’s notification sheet contains, in field 35, the question *“Does the patient possess any risk factors/comorbidities? If yes, which ones? (Mark x)”* followed by a list of 12 comorbidities to be measured, obesity being one of them^21^. After filling out this form, data are typed into the online information system. As a result of the format of the question in the notification sheet (marking “x” only for the comorbidities present), individuals with a marking on the variable “Obesity” with the category “Yes” were considered to have obesity, while individuals marked with the category “No” or with the field “Blank” were considered non-obese. Finally, individuals marked with the category “Ignored” were excluded from the analyses.

### Outcome

The outcome assessed was hospital death caused by COVID-19. The variable collected in the SIVEP Gripe contains three valid categories: hospital discharge (recovery)/death/death by other causes. Individuals who progressed to death by other causes (cases of severe COVID-19 whose subsequent death was not related to COVID-19) were excluded from the analytical sample, and the outcome was dichotomized into hospital discharge/death.

### Covariates Used in the Multivariable Adjustment

The demographic covariates gender (female/male) and self-declared skin color (White/Black/Mixed-race/Yellow/Indigenous/Not informed), postpartum women (no/yes), comorbidities at least weakly associated with hospital death caused by COVID-19 in the study sample (p < 0.20) (asthma, diabetes mellitus, chronic cardiovascular diseases, hematologic diseases, hepatic diseases, chronic neurological disease, chronic renal diseases, other pneumopathy, immunodeficiency diseases and Down syndrome), and the hospital region of residence were considered as potential confounders of the association between obesity and death.

In the initial stages of the pandemic response, the state was divided into 21 hospital regions to organize the referral network for the occupation of Intensive Care Unit (ICU) beds and for monitoring health system capacity. Considering the variability between regions, the COVID-19 region of residence was inserted into the multivariable adjustment with fixed effects.

The age range was evaluated as an effect modifier, stratified into four categories: 18–39 years, 40–59 years, 60–69 years, and 70 or more years.

### Statistical Analysis

The analytical sample was described with absolute and relative frequencies. Bivariate analysis (between the exposure and the covariates, and between the outcome and the covariates) was conducted using a chi-squared test of heterogeneity. A Poisson regression was employed to estimate crude and adjusted relative risks (RR), with 95% confidence intervals (95%CI). A likelihood ratio test was utilized to assess the level of statistical significance of the interaction term between obesity and age range. The multicollinearity of the final model was tested using the variance inflation factor.

A sensitivity analysis with model adjustment was conducted without including the comorbidities diabetes mellitus and chronic cardiovascular disease, which could function as mediators of the effect of obesity on the outcome, in order to evaluate their influence over the association of interest. To measure the impact of obesity over the hospital case-fatality rate in the population of adults hospitalized for COVID-19 in Rio Grande do Sul, we calculated the attributable fraction in the exposed — or with obesity (AF_o_) (AF_o_ = 1 - (1/RR)). Being this a census for all hospitalized cases for COVID-19 in the state, the prevalence of obesity of the sample, for each age range, was considered to be the prevalence in this hospitalized population (Pe), and the hospitalized population attributable risk $PAR={Pe}^{*}\frac{RR-1}{Pe*(RR-1)+1}$ , a measurement of the impact of obesity over the hospital case-fatality rate, was also calculated. Such measures aim to scale the impact of obesity as a prognostic factor in the group of cases of severe COVID-19. Moreover, the hospitalized PAR was calculated separately for Black people (those who declared themselves Black or Mixed-race) and other skin colors (White/Indigenous/Yellow) to observe the differential impact of obesity among Blacks and non-Blacks. In this analysis the category “not informed” for skin color was excluded. All analyses were conducted using Stata 14.0 software (Stata Corp., College Station, Texas, USA) and with a 95% significance level.

### Ethical Considerations

The secondary data utilized from SIVEP Gripe are de-identified and publicly available on the OpenDataSUS website^22^ maintained by the Ministry of Health. This study used aggregated data, with very little risk to participants.

## RESULTS

From the beginning of the COVID-19 pandemic until September 20, 2021, 108,293 hospitalizations for severe COVID-19 were recorded in the state of Rio Grande do Sul, Brazil. Among the 107,369 occurring in people aged 18 years or older, 98 progressed to death by other causes, 6,446 and 726 did not possess information on the studied outcome and exposure, respectively, on the date the database was accessed, leaving 100,099 hospitalizations in the analytical sample. Additionally, 38.98% of cases in the analytical sample were notified by public health facilities, 8.33% by private facilities, and 52.68% were notified by mixed health facilities (serving public and private).

The sample was mostly White (84.7%) and male (54.7%). Among the age strata generated by the analysis, 13.6% of the sample was between 18 and 39 years old, 36.7% between 40 and 59 years, 21.1% between 60 and 69 years, and 28.6% was 70 years or older. The most prevalent comorbidities were chronic cardiovascular disease (34.9%) and diabetes mellitus (24.0%) (Table 1).

**Table 1:** Characteristics of individuals aged 18 and over hospitalized for severe COVID-19 as of September 20, 2021, in the state of Rio Grande do Sul, Brazil. (n = 100,099).

Variables	Sample (total) n (%)	Age (ranges			

		18–39 (years) n (%)	40–59 (years) n (%)	60–69 (years) n (%)	70 (years or older) n (%)
Gender					
Female	45,380 (45.34)	6,024 (44.09)	15,376 (41.86)	9,515 (45.08)	14,465 (50.59)
Male	54,719 (54.66)	7,640 (55.91)	21,359 (58.14)	11,592 (54.92)	14,128 (49.41)
Skin color					
White	84,796 (84.71)	11,437 (83.70)	30,948 (84.25)	17,842 (84.53)	24,569 (85.93)
Black	4,054 (4.05)	623 (4.56)	1,485 (4.04)	906 (4.29)	1,040 (3.64)
Mixed-race	3,903 (3.90)	599 (4.38)	1,567 (4.27)	825 (3.91)	912 (3.19)
Yellow	258 (0.26)	52 (0.38)	93 (0.25)	47 (0.22)	66 (0.23)
Indigenous	173 (0.17)	37 (0.27)	59 (0.16)	35 (0.17)	42 (0.15)
Not informed	6,915 (6.91)	916 (6.70)	2,583 (7.03)	1,452 (6.88)	1,964 (6.87)
Asthma					
No	95,982 (95.89)	12,775 (93.49)	35,272 (96.02)	20,343 (96.38)	27,592 (96.50)
Yes	4,117 (4.11)	889 (6.51)	1,463 (3.98)	764 (3.62)	1,001 (3.50)
Diabetes					
No	76,066 (75.99)	12,791 (93.61)	30,002 (81.67)	14,106 (66.83)	19,167 (67.03)
Yes	24,033 (24.01)	873 (6.39)	6,733 (18.33)	7,001 (33.17)	9,426 (32.97)
Cardiovascular diseases					
No	65,160 (65.10)	12,485 (91.37)	27,431 (74.67)	11,861 (56.19)	13,383 (46.81)
Yes	34,939 (34.90)	1,179 (8.63)	9,304 (25.33)	9,246 (43.81)	15,210 (54.19)
Hematologic diseases					
No	99,327 (99.23)	13,595 (99.50)	36,536 (99.46)	20,933 (99.18)	28,263 (98.85)
Yes	772 (0.77)	69 (0.50)	199 (0.54)	174 (0.82)	330 (1.15)
Hepatic diseases					
No	98,945 (98.85)	13,595 (99.50)	36,342 (98.93)	20,757 (98.34)	28,251 (98.80)
Yes	1,154 (1.15)	69 (0.50)	393 (1.07)	350 (1.66)	342 (1.20)
Neurological diseases					
No	94,797 (94.70)	13,408 (98.13)	35,962 (97.90)	20,253 (95.95)	25,174 (88.04)
Yes	5,302 (5.30)	256 (1.87)	773 (2.10)	854 (4.05)	3,419 (11.96)
Chronic renal diseases					
No	96,315 (96.22)	13,455 (98.47)	35,826 (97.53)	20,147 (95.45)	26,887 (94.03)
Yes	3,784 (3.78)	209 (1.53)	909 (2.47)	960 (4.55)	1,706 (5.97)
Other pneumopathy					
No	94,882 (94.78)	13,488 (98.71)	35,932 (97.81)	19,701 (93.34)	25,761 (90.10)
Yes	5,217 (5.22)	176 (1.29)	803 (2.19)	1,406 (6.66)	2,832 (9.90)
Immunodeficiency diseases					
No	96,783 (96.69)	13,318 (97.47)	35,610 (96.94)	20,262 (96.00)	27,593 (96.50)
Yes	3,316 (3.31)	346 (2.53)	1,125 (3.06)	845 (4.00)	1,000 (3.50)
Postpartum women					
No	99,893 (99.79)	13,472 (98.59)	36,723 (99.97)	21,107 (100.00)	28,591 (99.99)
Yes	206 (0.21)	192 (1.41)	12 (0.03)	0 (0.00)	2 (0.01)
Down syndrome					
No	99,821 (99.72)	13,574 (99.34)	36,636 (99.73)	21,058 (99.77)	28,553 (99.86)
Yes	278 (0.28)	90 (0.66)	99 (0.27)	49 (0.23)	40 (0.14)
Total	100,099	13,664 (13.65)	36,735 (36.70)	21,107 (21.09)	28,593 (28.56)

The overall prevalence of obesity was 14.4%, 22.0% among individuals aged 18-39 years, 17.1% among individuals 40–59 years old, 13.7% among individuals aged 60–69 years, and 7.8% for those aged 70 and up. Women (16.5%), Black and Mixed-race people (18.7%), and those with the chronic conditions Down syndrome (28.8%), asthma (26.0%), and diabetes mellitus (18.9%) exhibited the highest prevalence (Table 2).

**Table 2:** Prevalence of obesity according to characteristics of individuals aged 18 and over hospitalized for severe COVID-19 as of September 20, 2021, in the state of Rio Grande do Sul, Brazil (n = 100,099).

Variables	Total sample % p	Age ranges				

		18–39 years % p	40–59 years % p	60–69 years % p	70 years or older % p	
Gender	< 0.001	< 0.001	< 0.001		< 0.001	< 0.001
Female	16.51	24.22	19.84		16.46	9.81
Male	12.65	20.20	15.14		11.46	5.77
Skin color	< 0.001	< 0.001	< 0.001		0.151	< 0.001
White	14.10	21.50	16.66		13.75	7.70
Black	18.55	29.37	22.02		15.56	9.71
Mixed-race	18.81	28.21	22.21		14.42	10.75
Yellow	15.12	19.23	13.98		12.77	15.15
Indigenous	13.29	5.41	25.42		8.57	7.14
Not informed	13.12	19.54	16.49		11.85	6.62
Asthma	< 0.001	< 0.001	< 0.001		< 0.001	< 0.001
No	13.90	21.17	16.57		13.34	7.54
Yes	25.97	33.41	30.01		23.56	15.28
Diabetes	< 0.001	< 0.001	< 0.001		< 0.001	< 0.001
No	12.97	20.73	15.00		11.10	5.96
Yes	18.94	40,09	26.48		18.97	11.57
Cardiovascular diseases	< 0.001	< 0.001	< 0.001		< 0.001	< 0.001
No	12.85	20.04	14.22		10.35	5.53
Yes	17.29	42.41	25.60		18.02	9.82
Hematologic diseases	0.850	0.529	0.712		0.043	0.282
No	14.40	21.99	17.10		13.67	7.79
Yes	14.64	18.84	18.09		18.97	9.39
Hepatic diseases	0.319	0.592	0.188		0,209	0.795
No	14.39	21.96	17.08		13.67	7.71
Yes	15.42	24.64	19.59		16.00	8.19
Neurological diseases	< 0.001	0.237	0.789		0.032	< 0.001
No	14.72	21.91	17.10		13.82	8.21
Yes	8.71	25.00	17.46		11.24	4.88
Chronic renal diseases	0.675	0.877	0.488		0,140	< 0.001
No	14.41	21.98	17.08		13.63	7.64
Yes	14.16	21.53	18.04		15.31	10.55
Other pneumopathy	0.016	0.038	< 0.001		0,305	0.001
No	14.46	21.89	16.94		13.78	7.64
Yes	13.26	28.41	24.41		12.80	9.39
Immunodeficiency diseases	< 0.001	< 0.001	< 0.001		0,157	0.115
No	14.54	22.18	17.29		13.78	7.86
Yes	10.34	13.87	11.38		12.07	6.50
Postpartum women	0.597	0.005	0,420		---	0.681
No	14.40	22.09	17.11		13.71	7.81
Yes	13.11	13.54	8.33		0.00	0.00
Down syndrome	< 0.001	0.001	0,059		0.001	0.004
No	14.36	21.87	17.09		13.67	7.80
Yes	28.78	36.67	24.24		30.61	20.00
Total	14.40	21.97	17.11		13.71	7.81

The overall in-hospital case fatality rate by COVID-19 was 33.1%, varying from 12.3% in 18-39-year-olds up to 53.9% in people aged 70 and older. Women had a slightly greater in-hospital case fatality rate (33.4%) compared to men (32.8%). Black people displayed the greatest in-hospital case fatality rate among all valid skin color categories. Individuals with several chronic conditions presented with a higher in-hospital case fatality rate. Those with neurological disease, who were generally of more advanced age and who possibly resided more frequently in long-term care institutions for older people, displayed the greatest in-hospital case fatality rate (60.0%) (Table 3).

**Table 3:** Case fatality rate for COVID-19 according to characteristics of individuals aged 18 years or older hospitalized for severe COVID-19 as of September 20, 2021, in the state of Rio Grande do Sul, Brazil (n = 100,099).

Variables	Total sample % p	Age ranges				

		18–39 years % p	40–59 years % p	60–69 years % p	70 years or older % p	
Gender	0.036	0.216	0,188		0.001	< 0.001
Female	33.39	12.65	22.23		36.49	51.86
Male	32.77	11.95	21.65		38.79	55.88
Skin color	< 0.001	< 0.001	< 0.001		< 0.001	< 0.001
White	32.32	11.71	21.28		36.45	52.83
Black	38.21	15.25	25.93		47.46	61.44
Mixed-race	35.95	17.20	26.61		43.15	57.79
Yellow	36.43	19.23	20.43		57.45	57.58
Indigenous	28.90	5.41	13.56		40.00	61.90
Not informed	37.30	13.76	24.35		43.87	60.44
Asthma	< 0.001	0.004	0.085		0.345	0.003
No	33.16	12.05	21.82		37.81	54.01
Yes	30.41	15.30	23.72		36.13	49.25
Diabetes	< 0.001	< 0.001	< 0.001		< 0.001	< 0.001
No	29.03	11.16	19.62		33.97	52.06
Yes	45.76	28.41	32.02		45.36	57.48
Cardiovascular diseases	< 0.001	< 0.001	< 0.001		< 0.001	< 0.001
No	27.28	11.22	19.60		34.69	51.42
Yes	43.82	23.24	28.67		41.67	55.98
Hematologic diseases	< 0.001	< 0.001	< 0.001		< 0.001	0.001
No	32.89	12.14	21.78		37.59	53.74
Yes	54.02	36.23	42.21		57.47	63.03
Hepatic diseases	< 0.001	< 0.001	< 0.001		< 0.001	< 0.001
No	32.79	12.13	21.65		37.38	53.68
Yes	55.63	37.68	44.78		60.00	67.25
Neurological diseases	< 0.001	< 0.001	< 0.001		< 0.001	< 0.001
No	31.54	11.87	21.47		37.12	51.93
Yes	59.98	32.81	41.53		52.81	67.97
Chronic renal diseases	< 0.001	< 0.001	< 0.001		< 0.001	< 0.001
No	32.11	11.89	21.38		36.89	52.93
Yes	57.06	35.89	42.13		55.73	68.35
Other pneumopathy	< 0.001	< 0.001	< 0.001		< 0.001	< 0.001
No	31.85	12.09	21.54		36.76	52.80
Yes	54.92	25.00	37.61		51.64	63.31
Immunodeficiency diseases	< 0.001	< 0.001	< 0.001		< 0.001	< 0.001
No	32.30	11.53	21.21		36.83	53.32
Yes	54.79	40.46	43.47		59.76	68.30
Postpartum women	< 0.001	0.061	0.661		---	0.127
No	33.08	12.20	21.90		37.75	53.85
Yes	16.50	16.67	16.67		0.00	0.00
Down syndrome	0.039	< 0.001	< 0.001		0,185	0.864
No	33.03	12.10	21.84		37.77	53.85
Yes	39.21	36.67	41.41		28.57	52.50
Total	33.05	12.26	21.89		37.75	53.85

The multivariate analysis found the covariate age range to modify the effect of obesity over the in-hospital case fatality rate (p < 0.001 for interaction). The younger the age stratum, the greater the strength of association. For the 18–39 years range, people with obesity exhibited a 2.54 (95%CI: 2.33–2.77) times greater risk of death when compared to people without obesity. Individuals 70 years or older and with obesity displayed a 9% (RR = 1.09; 95%CI: 1.05–1.13) greater risk of death compared to people without obesity in the same age group (Table 4). There was no evidence of collinearity in the covariates studied.

**Table 4:** Crude and adjusted analyses for association between obesity and the case fatality rate due to COVID-19 in individuals aged 18 years or more hospitalized for severe COVID-19 in the state of Rio Grande do Sul, Brazil.

Variable	Crude RR	CI95%	p	Adjusted^a^ RR	CI95%	p
	**18–39 years**					
Obesity			< 0.001			< 0.001
No	Ref	2.44–2.91		Ref	2.33–2.77	
Yes	2.66			2.54		
	**40–59 years**					
Obesity			< 0.001			< 0.001
No	Ref			Ref		
Yes	1.84	1.76–1.91		1.74	1.67–1.81	
			**60–69 years**			
Obesity				< 0.001		< 0.001
No	Ref			Ref		
Yes	1.36	1.3 –1.42		1.34	1.28–1.39	
	**70 years or older**					
Obesity			< 0.001			< 0.001
No	Ref			Ref		
Yes	1.10	1.06–1.14		1.09	1.05–1.13	

^a^ Adjusted for gender, self-declared skin color, asthma, diabetes mellitus, chronic cardiovascular diseases, hematologic diseases, hepatic diseases, chronic neurological. disease, chronic renal diseases, other pneumopathy, Immunodeficiency diseases, postpartum women and Down syndrome, and the region of residence of the individual.

Table 5 displays the attributable fraction among the exposed (AF_o_) of obesity for the outcome of death among adults hospitalized for severe COVID-19 and the attributable risk of obesity for the outcome of death in the overall population of adults hospitalized with severe COVID-19 (hospitalized PAR). For people with obesity at the time of hospitalization, 60.6% of deaths by COVID-19 in individuals 18–39 years old and 42.5% of deaths by COVID-19 in individuals 40–59 years old could have been prevented if obesity were eliminated. For all hospitalizations, both with and without obesity, the hospitalized PAR of obesity for individuals 18–39 years old was 25.3%, meaning that one-fourth of deaths among all individuals 18-39 years old who were hospitalized for COVID-19 could have been avoided if obesity were eliminated. Meanwhile, for the 40-59-year-old age range, the hospitalized PAR was 11.2%. Since the covariate skin color did not modify the effect of obesity on the in-hospital case-fatality rate (likelihood ratio test for interaction: p-value = 0.33) and the prevalence of obesity among Black and Mixed-race people hospitalized for COVID-19 was greater than that of the remaining skin color groups, Table 5 also presents the hospitalized PARs calculated separately for Black people (Black or Mixed-race skin color) and for the remaining skin color categories (White/Indigenous/Yellow). Obesity was found to have a larger impact on the hospital case-fatality rate by COVID-19 among young Black people.

**Table 5:** Attributable fraction among the exposed/with obesity (AFo), hospitalized population attributable risk (hospitalized PAR) in the adult population hospitalized for COVID-19 and among Black and non-Black people in the same population in the state of Rio Grande do Sul, Brazil.

Age range	AF_o_ in %	Hospitalized PAR in %		Hospitalized PAR in %		

				Black		Non-Black
18–39 years	60.63	25.28		31.73	24.82	
40–59 years	42.53	11.24		14.07	10.98	
60–69 years	25.37	4.45		4.86	4.46	
70 years or older	8.26	0.70		0.91	0.69	

In the sensitivity analysis, the covariates diabetes mellitus and chronic cardiovascular disease were excluded from the final model for being possible mediators of the association of interest and due to the fact that the cross-sectional nature of the data on comorbidities did not allow for the temporality or the directionality of the association between the different comorbidities recorded at the time of hospitalization to be established. In the absence of adjustment for diabetes mellitus and chronic cardiovascular disease, the magnitude of the effect of obesity on death increased only slightly. For the 18−39-year-old age range, people with obesity displayed a risk 2.59 (95%CI: 2.38–2.83) times greater of death when compared to people without obesity. Individuals 70 years or older and with obesity presented with a 14% (RR = 1.14; 95%CI: 1.09–1.18) greater risk of death compared to those without obesity in the same age group.

## DISCUSSION

This study estimated the relative risk associated with obesity and the impact of this chronic condition on the hospital case-fatality rate due to COVID-19 in the adult population hospitalized for the disease in the state of Rio Grande do Sul, for the different demographic groups analyzed. The relative risk associated with obesity was notably higher for the younger age groups. Additionally, the prevalence of obesity was higher for the younger age ranges and for Black people, resulting in an impact estimate that pointed out that, in the hospitalized population aged 18−39 years, obesity was responsible for more than one-fourth of total deaths, with a hospitalized PAR of 31.7% for Black people and 24.8% for non-Black people.

Obesity alters pulmonary function via mechanic and inflammatory mechanisms, elevating the risk of displaying symptoms and progressing to more severe cases of respiratory failure for people with obesity^23^. Exposed individuals have chronically greater concentrations of leptin and smaller concentrations of adiponectin, leading to dysregulation of the immune system^24^. In addition, individuals with obesity present with larger concentrations of various pro-inflammatory cytokines, such as TNF-α, MCP-1 and IL-6, which affect immunity^25^ and can exacerbate the inflammatory process in airways present in cases of severe COVID-19.

Obesity has been reported as the most common underlying condition for patients with COVID-19 younger than 64 years^26^. In addition, it frequently co-occurs with other chronic conditions. Studies evaluating the relationship between obesity and death by COVID-19^11^ found association even without reporting an interaction with age. Hendren et al.^17^ (2021) analyzed data of patients hospitalized with COVID-19 in 88 hospitals in the U.S. and found the association with death to be stronger than that of body-mass-index (BMI) in adults younger than 50 years, intermediate in adults aged 51–70 years and weaker in adults older than 70 years, corroborating the results of our study. Also, Klang et al.^16^ (2020) estimated a strong association between BMI and death in individuals younger than 50 years, and a weaker though still significant association among patients aged 50 years and older.

The National Health Survey 2019 conducted in Brazil estimated that one-fourth of the Brazilian adult population had obesity. In the general population, those aged 18−24 years had less obesity (10.7%) while individuals aged 40–59 years had the most obesity (34.4%), followed by those aged 60 years and above (24.8%)^9^. However, in the hospitalized population analyzed, younger patients had more obesity. Although the youngest age group displayed a smaller risk of death by COVID-19, obesity is an extremely important prognostic factor of death in this age range.

The larger prevalence of obesity found in Black people could be due to the accumulation of adverse circumstances associated with their overall lower socioeconomic position, such as poorer access to healthy foods and less time available to practice physical activity. A study by De Azevedo Barros et al.^27^ (2016) evaluated social inequalities in health behaviors among Brazilian adults and found a greater prevalence of smoking, physical inactivity during leisure, sedentary lifestyles, whole milk consumption and low consumption of leafy greens, vegetables, and fruits in the non-White population. Therefore, to improve indicators of chronic conditions such as obesity, it is necessary to reduce inequalities implementing population-level diet and exercise promotion-oriented interventions. Regular monitoring of nutrition data disaggregated by population subgroups such as wealth quintiles or deciles as well as skin color/ethnicity is also essential to track progress and guide public health actions with an equity lens^18^.

In Brazil, the Strategic Action Plan for Tackling Chronic Non-Communicable Diseases including obesity serves as a guideline for the prevention of risk factors for Non-Communicable Diseases and for population health promotion, with the aim of reducing health inequalities^28^. In this way, it will be important to implement the Global Strategy for Diet, Physical Activity and Health^29^ to stop the increase in obesity. Our results are important for supporting the actions outlined in this strategic plan. Other actions to reduce the burden of disease caused by obesity are globally necessary, with a focus on youth and Black people in middle-income countries.

Our results must be interpreted considering certain limitations. Obesity as a variable was primarily collected from routine medical records from patients in health facilities in Rio Grande do Sul that received patients with severe COVID-19. It was not possible to describe the form of recording/collection conducted by each health facility. The variability in the measurement of the exposure tends to generate non-differential classification error, potentially underestimating the estimates of relative risk. The estimates of AF_o_ and hospitalized PAR may also have been underestimated, in the event that the prevalence of obesity was underestimated. Nevertheless, the results show a sufficiently strong effect to justify the study’s conclusions of the elevated impact of obesity and of the groups for which there is a greater attributable risk. Another limitation related to the measurement of the exposure obesity was that the availability of the variable in dichotomic form precluded a dose-response analysis of the effect of increased BMI on relative risk of death. In addition, unfortunately, the variable level of schooling exhibited 57% missing data and was not included in the multivariate analysis for this reason. However, the variable skin color is a strong proxy for socioeconomic position in Brazil. There was a very discreet change in results from the restricted sample to when level of schooling was included in the multivariate model (data not shown).

This study also displays important strengths. First, we analyzed all recorded cases of admissions for severe COVID-19 in public and private health facilities in the state, resulting in a representative sample with maximum statistical power to identify existent associations. Second, we conducted an analysis of effect modification that was barely susceptible to exposure measurement error and, to the best of our knowledge, this study is the first to estimate the proportion of deaths that could be avoided among hospitalized people if obesity were completely prevented. In the sensitivity analysis there were no important differences observed in the effects of obesity when cardiovascular diseases and diabetes mellitus were removed from the final model, which probably indicates that the inclusion of other unanalyzed comorbidities would not significantly alter the estimates obtained.

## CONCLUSIONS

Obesity had a large impact on the in-hospital case-fatality rate due to COVID-19 among young adults who were hospitalized. Black people generally suffered a greater impact for displaying a higher prevalence of obesity in the study sample. The data highlight the magnitude of the risk attributable to obesity, a key chronic condition. In the context of the pandemic, prioritization of care measures that prevent contagion and of interventions for people with obesity have great potential for effectiveness in reducing years of life lost to COVID-19.
